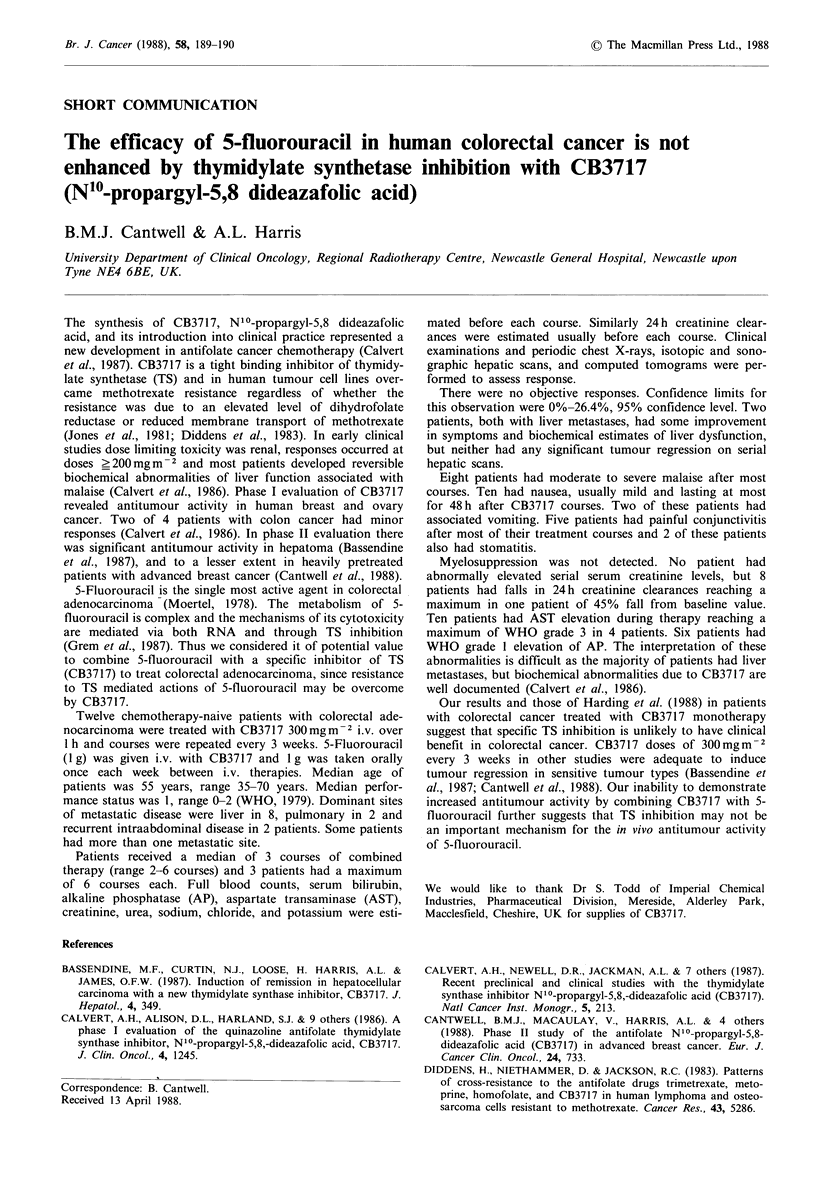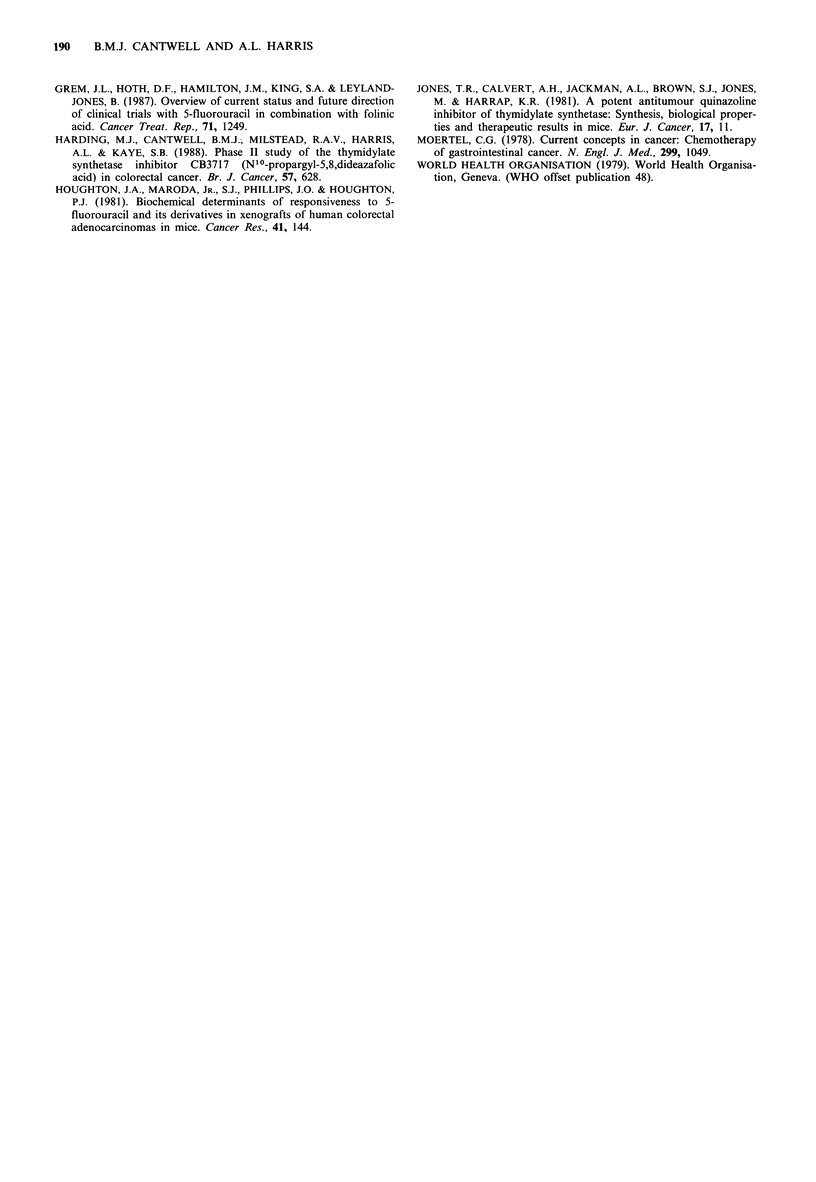# The efficacy of 5-fluorouracil in human colorectal cancer is not enhanced by thymidylate synthetase inhibition with CB3717 (N10-propargyl-5,8 dideazafolic acid).

**DOI:** 10.1038/bjc.1988.190

**Published:** 1988-08

**Authors:** B. M. Cantwell, A. L. Harris

**Affiliations:** University Department of Clinical Oncology, Newcastle General Hospital, Newcastle upon Tyne, UK.


Br. J. Cancer (1988), 58, 189–190 © The Macmillan Press Ltd., 1988

SHORT COMMUNICATION

### The efficacy of 5-fluorouracil in human colorectal cancer is not enhanced by thymidylate synthetase inhibition with CB3717 ($N^{10}$-propargyl-5,8 dideazafolic acid)

B.M.J. Cantwell & A.L. Harris

*University Department of Clinical Oncology, Regional Radiotherapy Centre, Newcastle General Hospital, Newcastle upon Tyne NE4 6BE, UK.*

The synthesis of CB3717, $N^{10}$-propargyl-5,8 dideazafolic acid, and its introduction into clinical practice represented a new development in antifolate cancer chemotherapy (Calvert *et al.*, 1987). CB3717 is a tight binding inhibitor of thymidylate synthetase (TS) and in human tumour cell lines overcame methotrexate resistance regardless of whether the resistance was due to an elevated level of dihydrofolate reductase or reduced membrane transport of methotrexate (Jones *et al.*, 1981; Diddens *et al.*, 1983). In early clinical studies dose limiting toxicity was renal, responses occurred at doses $\geq 200\,mg\,m^{-2}$ and most patients developed reversible biochemical abnormalities of liver function associated with malaise (Calvert *et al.*, 1986). Phase I evaluation of CB3717 revealed antitumour activity in human breast and ovary cancer. Two of 4 patients with colon cancer had minor responses (Calvert *et al.*, 1986). In phase II evaluation there was significant antitumour activity in hepatoma (Bassendine *et al.*, 1987), and to a lesser extent in heavily pretreated patients with advanced breast cancer (Cantwell *et al.*, 1988).

5-Fluorouracil is the single most active agent in colorectal adenocarcinoma (Moertel, 1978). The metabolism of 5-fluorouracil is complex and the mechanisms of its cytotoxicity are mediated via both RNA and through TS inhibition (Grem *et al.*, 1987). Thus we considered it of potential value to combine 5-fluorouracil with a specific inhibitor of TS (CB3717) to treat colorectal adenocarcinoma, since resistance to TS mediated actions of 5-fluorouracil may be overcome by CB3717.

Twelve chemotherapy-naive patients with colorectal adenocarcinoma were treated with CB3717 $300\,mg\,m^{-2}$ i.v. over 1 h and courses were repeated every 3 weeks. 5-Fluorouracil (1 g) was given i.v. with CB3717 and 1 g was taken orally once each week between i.v. therapies. Median age of patients was 55 years, range 35–70 years. Median performance status was 1, range 0–2 (WHO, 1979). Dominant sites of metastatic disease were liver in 8, pulmonary in 2 and recurrent intraabdominal disease in 2 patients. Some patients had more than one metastatic site.

Patients received a median of 3 courses of combined therapy (range 2–6 courses) and 3 patients had a maximum of 6 courses each. Full blood counts, serum bilirubin, alkaline phosphatase (AP), aspartate transaminase (AST), creatinine, urea, sodium, chloride, and potassium were estimated before each course. Similarly 24 h creatinine clearances were estimated usually before each course. Clinical examinations and periodic chest X-rays, isotopic and sonographic hepatic scans, and computed tomograms were performed to assess response.

There were no objective responses. Confidence limits for this observation were 0%–26.4%, 95% confidence level. Two patients, both with liver metastases, had some improvement in symptoms and biochemical estimates of liver dysfunction, but neither had any significant tumour regression on serial hepatic scans.

Eight patients had moderate to severe malaise after most courses. Ten had nausea, usually mild and lasting at most for 48 h after CB3717 courses. Two of these patients had associated vomiting. Five patients had painful conjunctivitis after most of their treatment courses and 2 of these patients also had stomatitis.

Myelosuppression was not detected. No patient had abnormally elevated serial serum creatinine levels, but 8 patients had falls in 24 h creatinine clearances reaching a maximum in one patient of 45% fall from baseline value. Ten patients had AST elevation during therapy reaching a maximum of WHO grade 3 in 4 patients. Six patients had WHO grade 1 elevation of AP. The interpretation of these abnormalities is difficult as the majority of patients had liver metastases, but biochemical abnormalities due to CB3717 are well documented (Calvert *et al.*, 1986).

Our results and those of Harding *et al.* (1988) in patients with colorectal cancer treated with CB3717 monotherapy suggest that specific TS inhibition is unlikely to have clinical benefit in colorectal cancer. CB3717 doses of $300\,mg\,m^{-2}$ every 3 weeks in other studies were adequate to induce tumour regression in sensitive tumour types (Bassendine *et al.*, 1987; Cantwell *et al.*, 1988). Our inability to demonstrate increased antitumour activity by combining CB3717 with 5-fluorouracil further suggests that TS inhibition may not be an important mechanism for the *in vivo* antitumour activity of 5-fluorouracil.

We would like to thank Dr S. Todd of Imperial Chemical Industries, Pharmaceutical Division, Mereside, Alderley Park, Macclesfield, Cheshire, UK for supplies of CB3717.

### References

BASSENDINE, M.F., CURTIN, N.J., LOOSE, H. HARRIS, A.L. & JAMES, O.F.W. (1987). Induction of remission in hepatocellular carcinoma with a new thymidylate synthase inhibitor, CB3717. *J. Hepatol.*, 4, 349.

CALVERT, A.H., ALISON, D.L., HARLAND, S.J. & 9 others (1986). A phase I evaluation of the quinazoline antifolate thymidylate synthase inhibitor, $N^{10}$-propargyl-5,8,-dideazafolic acid, CB3717. *J. Clin. Oncol.*, 4, 1245.

Correspondence: B. Cantwell.
Received 13 April 1988.

CALVERT, A.H., NEWELL, D.R., JACKMAN, A.L. & 7 others (1987). Recent preclinical and clinical studies with the thymidylate synthase inhibitor $N^{10}$-propargyl-5,8,-dideazafolic acid (CB3717). *Natl Cancer Inst. Monogr.*, 5, 213.

CANTWELL, B.M.J., MACAULAY, V., HARRIS, A.L. & 4 others (1988). Phase II study of the antifolate $N^{10}$-propargyl-5,8-dideazafolic acid (CB3717) in advanced breast cancer. *Eur. J. Cancer Clin. Oncol.*, 24, 733.

DIDDENS, H., NIETHAMMER, D. & JACKSON, R.C. (1983). Patterns of cross-resistance to the antifolate drugs trimetrexate, metoprine, homofolate, and CB3717 in human lymphoma and osteosarcoma cells resistant to methotrexate. *Cancer Res.*, 43, 5286.

GREM, J.L., HOTH, D.F., HAMILTON, J.M., KING, S.A. & LEYLAND-JONES, B. (1987). Overview of current status and future direction of clinical trials with 5-fluorouracil in combination with folinic acid. *Cancer Treat. Rep.*, **71**, 1249.

HARDING, M.J., CANTWELL, B.M.J., MILSTEAD, R.A.V., HARRIS, A.L. & KAYE, S.B. (1988). Phase II study of the thymidylate synthetase inhibitor CB3717 ($N^{10}$-propargyl-5,8,dideazafolic acid) in colorectal cancer. *Br. J. Cancer*, **57**, 628.

HOUGHTON, J.A., MARODA, JR., S.J., PHILLIPS, J.O. & HOUGHTON, P.J. (1981). Biochemical determinants of responsiveness to 5-fluorouracil and its derivatives in xenografts of human colorectal adenocarcinomas in mice. *Cancer Res.*, **41**, 144.

JONES, T.R., CALVERT, A.H., JACKMAN, A.L., BROWN, S.J., JONES, M. & HARRAP, K.R. (1981). A potent antitumour quinazoline inhibitor of thymidylate synthetase: Synthesis, biological properties and therapeutic results in mice. *Eur. J. Cancer*, **17**, 11.

MOERTEL, C.G. (1978). Current concepts in cancer: Chemotherapy of gastrointestinal cancer. *N. Engl. J. Med.*, **299**, 1049.

WORLD HEALTH ORGANISATION (1979). World Health Organisation, Geneva. (WHO offset publication 48).